# Effects of Long Non-Coding RNAs Induced by the Gut Microbiome on Regulating the Development of Colorectal Cancer

**DOI:** 10.3390/cancers14235813

**Published:** 2022-11-25

**Authors:** Shiying Fan, Juan Xing, Zhengting Jiang, Zhilin Zhang, Huan Zhang, Daorong Wang, Dong Tang

**Affiliations:** 1 Clinical Medical College, Yangzhou University, Yangzhou 225000, China; 2 Department of General Surgery, Institute of General Surgery, Clinical Medical College, Northern Jiangsu People’s Hospital, Yangzhou University, Yangzhou 225000, China

**Keywords:** colorectal cancer, lncRNAs, gut microbiome, regulation

## Abstract

**Simple Summary:**

The gut microbiome can regulate the long non-coding RNAs (lncRNAs) expression, which subsequently impacts the host transcriptome to change the expression of downstream target molecules, ultimately resulting in the development and progression of colorectal cancer (CRC). We focused on the important role of the microbiome in CRC and their effects on CRC-related lncRNAs. The aim of our study was to illustrate the mechanisms by which the gut microbiome mediated the occurrence and progression of CRC through the regulation of lncRNAs, because the regulatory role of the gut microbiome-induced lncRNAs in CRC has great potential and may serve as the foundation for future clinical CRC treatment, such as probiotic supplements and lncRNAs intervention.

**Abstract:**

Although an imbalanced gut microbiome is closely associated with colorectal cancer (CRC), how the gut microbiome affects CRC is not known. Long non-coding RNAs (lncRNAs) can affect important cellular functions such as cell division, proliferation, and apoptosis. The abnormal expression of lncRNAs can promote CRC cell growth, proliferation, and metastasis, mediating the effects of the gut microbiome on CRC. Generally, the gut microbiome regulates the lncRNAs expression, which subsequently impacts the host transcriptome to change the expression of downstream target molecules, ultimately resulting in the development and progression of CRC. We focused on the important role of the microbiome in CRC and their effects on CRC-related lncRNAs. We also reviewed the impact of the two main pathogenic bacteria, *Fusobacterium nucleatum* and enterotoxigenic *Bacteroides fragilis*, and metabolites of the gut microbiome, butyrate, and lipopolysaccharide, on lncRNAs. Finally, available therapies that target the gut microbiome and lncRNAs to prevent and treat CRC were proposed.

## 1. Introduction

Colorectal cancer (CRC) is the second most common cause of cancer-related death worldwide and the third most prevalent cancer [1]. The etiology of CRC is complex; nonetheless, ecological dysbiosis of the gut microbiome has been proven to play a significant role in the disease’s progression [2,3]. The human gut contains a variety of bacteria called the gut microbiome, which is crucial to the host’s immune system and metabolism [4]. The gut microbiome breaks down indigestible dietary components, synthesizes vitamins and other nutrients, detoxifies metabolic metabotypes, regulates the immune response, sends signals for the maintenance of mucosal integrity and renewal of epithelial cells, and secretes antimicrobial substances, which is helpful for the normal function of the colon [5]. Disorders of the immune system and metabolism lead to discomfort and even diseases. Therefore, dysbiosis of the gut microbiome may lead to the physiological functions of the organism malfunctioning and hence cause disease [6]. However, the processes through which the gut microbiota mediates the initiation and progression of CRC are not yet fully understood.

Recent studies have revealed that abnormal expressions of long non-coding RNAs (lncRNAs), which are RNAs longer than 200 nucleotides and without the ability to code for proteins, are linked to the occurrence, progression, recurrence, metastasis, and chemoresistance of CRC, which can assist in diagnosis, determining prognosis, and providing therapeutic targets in CRC [7,8,9,10]. The lncRNAs are often expressed at lower levels but show more cell type-specific expression patterns than protein-coding genes [11]. The lncRNAs regulate many key cellular activities, including cell proliferation, apoptosis, differentiation, and metabolism [12]. In addition, lncRNAs regulate physiological and pathological processes such as cell cycle and DNA damage repair, whose disruption can result in the progression of malignant tumors [13,14]. Furthermore, lncRNAs can control the pathogenesis of CRC from a variety of angles, primarily involving the tumor microenvironment (TME) that includes inflammation, immune evasion and exosome secretion, epithelial-mesenchymal transition (EMT), stemness maintenance, and angiogenesis of tumor, thus allowing them to control the occurrence, development, and treatment of CRC [15].

One study found only low levels of overlap in the expressions of lncRNAs in the colon of mice colonized with specific different gut microbes compared to germ-free mice, and most of the changed lncRNAs were type-specific, revealing that the expression of lncRNAs was altered differently depending on the type of bacterial colonization [16]. This finding raised the possibility of the influence of the gut microbiome on the expressions of lncRNAs, which in turn can target their downstream genes and activate signaling pathways, like Wnt and nuclear factor kappa-B (NF-κB), thereby influencing the course of CRC development [8,17]. However, due to limited knowledge about the connections between the multiple distinct lncRNAs and the gut microbiota, it is difficult to study all potential pairwise regulatory interactions between lncRNAs and the gut microbiome. There is a need to research the regulatory relationship between the gut microbiota and lncRNAs since potential associations between different lncRNAs and the gut microbiome of CRC patients may be candidates for functional inspection. In this review, we outlined the mechanisms by which the gut microbiome mediated the occurrence and progression of CRC through the regulation of lncRNAs, thus raising the idea that CRC might be treated and prevented by applying probiotics and fecal transplantation as preventative measures. The processes of gut microbes and their metabolites influencing the development of CRC through the control of lncRNAs are listed in Supplementary Table S1.

## 2. Biogenesis and the Mode of Action of lncRNAs

**Figure 1:**
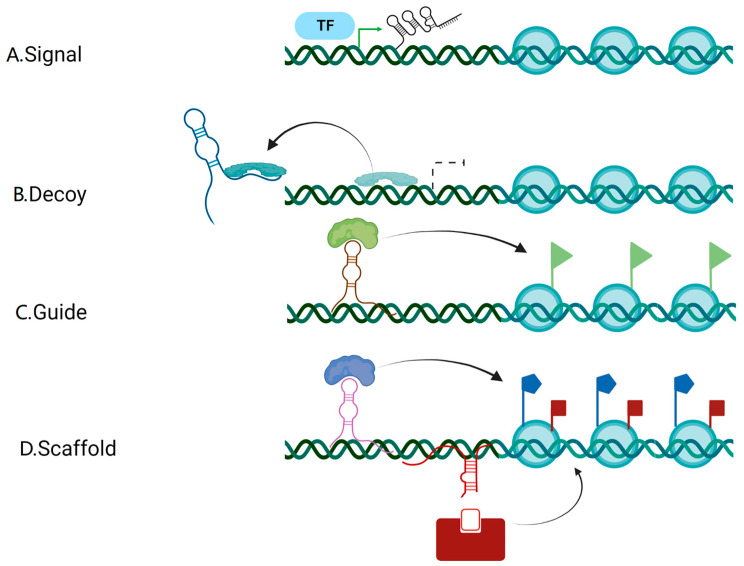
Biogenesis and the mode of action of lncRNAs. (**A**) Signal: some lncRNAs are specifically transcribed under different TFs and signaling pathways, and they subsequently participate in specific signaling pathways as signaling molecules. (**B**) Decoy: a certain type of lncRNAs are transcribed and bind directly to protein targets but are inactive, thus inhibiting the function of the molecule and the signaling pathway. (**C**) Guidance: certain lncRNAs, when bound to proteins, can direct the localization of ribonucleoprotein complex to specific targets (colored flags), thereby regulating the transcription of downstream molecules. (**D**) Scaffolding: some lncRNAs act as a “central platform” to bring multiple proteins together to form ribonucleoprotein complexes, enabling the intersection and integration of information between various signaling pathways and facilitating the rapid feedback and regulation of the body or cell to external signals and stimuli. (Created with BioRender.com). lncRNAs: long non-coding RNAs; TFs: transcription factors.

Most lncRNAs are transcriptionally regulated by RNA polymerase II (Pol II), demanding the insertion of a 3’ end poly (A) tail and a 5’ end m7G cap [18,19]. Most lncRNAs are exported and localized in the cytoplasm, except a small number is kept in the nucleus [20]. Due to the limited number of exons contained in the sequence, the export of lncRNAs mainly depends on the nuclear RNA export factor 1 (NXF1) pathway [21]. The lncRNAs can exist in various forms and perform appropriate activities once they enter the cytoplasm [20]. Previous studies have confirmed that the mode of action of lncRNAs can be divided into four categories: signal, decoy, guide, and scaffold [22,23] (Figure 1). The signal mode means that some lncRNAs are specifically transcribed under different activation conditions and signaling pathways, and they subsequently participate in specific signaling pathways as signaling molecules; the decoy mode refers to a certain type of lncRNAs that are transcribed and bind directly to protein targets but are inactive, thus inhibiting the function of the molecule and the signaling pathway; the guidance mode means that certain lncRNAs, when bound to proteins, can target ribonucleoprotein complexes to specific targets, thereby regulating the transcription of downstream molecules; the scaffolding mode describes how some lncRNAs act as a “central platform” to bring multiple proteins together to form ribonucleoprotein complexes, enabling the intersection and integration of information between various signaling pathways and facilitating the rapid feedback and regulation of the body or cell to external signals and stimuli. Of note, a single lncRNA may accord with several prototypes, and as such, each prototype is not intended to be mutually exclusive [22]. Thus, it is evident that lncRNAs have a complex mode of action and that their abnormal expressions will change several crucial cellular processes.

Overall, the expression of lncRNAs is tightly regulated in normal cells; however, abnormal expression of lncRNAs is present in tumor cells. A growing body of evidence states that numerous lncRNAs with aberrant expression, including HOTAIR, CCAT1, and LINC0065, are found in CRC [24,25,26]. Based on the aforementioned complex biogenesis and molecular regulation mechanisms, the abnormally expressed lncRNAs play a significant role in regulating many signaling pathways, such as Wnt and NF-κB, thus affecting various aspects of CRC occurrence, development, diagnosis, and prognosis [8,27]. The various pathophysiological mechanisms of CRC mediated by aberrantly produced lncRNAs offer a fresh viewpoint for research on the disease.

## 3. The Development of CRC Promoted by the Disturbance of the Gut Microbiome 

Dysbiosis of the gut microbiome is associated with various illnesses, including autoimmune, metabolic, and neurological disorders and cancer [28]. Enrichment of pathogenic bacteria in the gut can cause DNA damage, promote inflammation, induce tumor cell proliferation, and shield the tumor from immune attack [29]. Intestinal ecological dysregulation has been identified in several macrogenomic studies on CRC as a crucial risk factor for the development of colorectal malignancies [30,31,32]. The Alpha-bugs and the bacterial driver-passenger models are widely accepted theories for how dysregulated gut microbiota triggers the development and progression of CRC [33,34]. According to the Alpha-bugs model, on the one hand, the gut microbiome directly causes intestinal epithelial cell carcinogenesis through the secretion of toxic proteins, and on the other, changes in the gut microbiome cause abnormal mucosal immune responses that allow the accumulation of cancerous intestinal epithelial cells, and subsequently CRC [33]. According to the bacterial driver-passenger model, pathogenic bacteria are called “bacterial drivers”. Bacterial drivers cause DNA damage, induce inflammation in the intestinal epithelium, and are the first to change the intestinal microenvironment as CRC initiators, making it possible for opportunistic bacteria (bacterial passengers) to have a survival advantage in the microenvironment and promote CRC development [34,35]. Nevertheless, the fundamental mechanism is not well understood.

The underlying processes by which the gut microbiome contributes to CRC have been the subject of various research, which have shown that numerous pathogenic bacteria and metabolites of the gut microbiome can affect the progression of CRC by controlling the expression of lncRNAs [17,36,37]. By upregulating LINC00152, a gene associated with intestinal inflammation, cancer cell migration, and invasion, Wang et al. discovered that *Salmonella typhimurium* infection affected the lncRNAs expression in intestinal epithelial cells [36]. According to Chen et al., *Fusobacterium nucleatum* infection upregulates the lncRNAs Keratin7-antisense (KRT7-AS) and Keratin7 (KRT7) by triggering the NF-κB pathway to promote CRC metastasis [17]. Conversely, the gut microbiome metabolite butyrate-induced lncRNA LncLy6C suppressed intestinal inflammation, thus lowering the risk of CRC, according to the research by Gao et al. [37]. Therefore, it is reasonable to assume that gut microbiome disorders can influence CRC development via mediating lncRNAs. These studies stated the mechanisms by which a dysfunctional gut microbiome promotes CRC and the influence of the gut microbiome on the expression of lncRNAs, thus suggesting that lncRNAs play a non-negligible role in CRC induced by the dysfunctional gut microbiome.

## 4. Microbial Regulation of CRC Mediated by lncRNAs

### 4.1. Fusobacterium Nucleatum (F. nucleatum) Promotes CRC Metastasis as Well as Enhances Drug Resistance by Regulating lncRNAs

**Figure 2:**
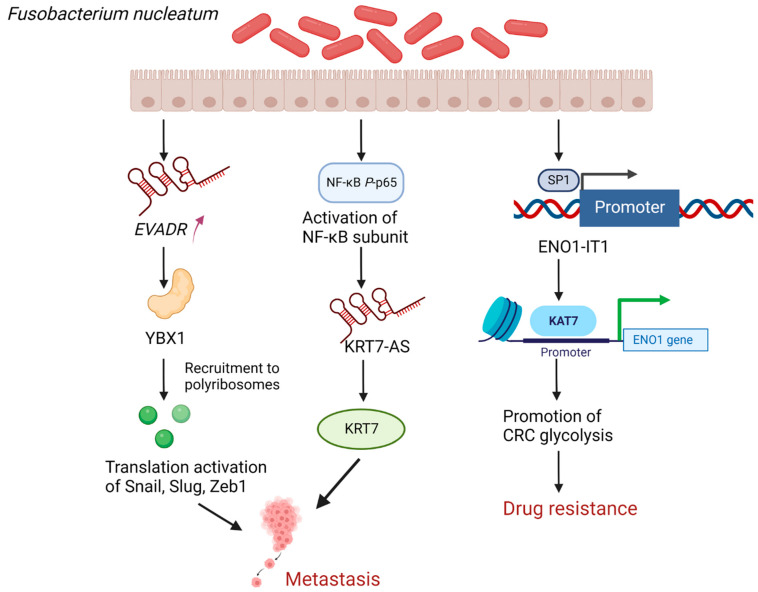
*F. nucleatum* promotes CRC metastasis as well as enhances drug resistance by upregulating *EVADR*, KRT7-AS, and ENO1-IT1. Elevated *EVADR* directs RBP YBX1 to recruit EMT-related factors, including Snail, Slug, and Zeb1, to polyribosomes, and enhances the translation of these EMT-related factors, thereby inducing EMT, ultimately facilitating colorectal cancer metastasis. *F. nucleatum* infection activates the NF-κB pathway, and activated NF-κB *P*-p65 (activated NF-κB subunit) may upregulate KRT7-AS by increasing the transcriptional activity of KRT7-AS, then activating the downstream target of KRT7-AS, KRT7, which regulates CRC metastasis. *F. nucleatum* upregulates the binding efficiency of the transcription factor SP1 to the promoter region of lncRNA ENO1-IT1 to activate the transcription of lncRNA ENO1- IT1 and subsequently recruit KAT7 to the promoter of the ENO1 gene to regulate ENO1 transcription via epigenetic modulation, thereby activating CRC glycolysis, which enhances the drug resistance. (Created with BioRender.com). *F. nucleatum*: *Fusobacterium nucleatum*; EVADR: endogenous retroviral-associated adenocarcinoma lncRNA; KRT7-AS: Keratin7-antisense; ENO1-IT1: enolase1-intronic transcript 1; RBP: RNA-binding protein; KRT7: Keratin7; EMT: epithelial mesenchymal transition; lncRNA: long non-coding RNA.

*F. nucleatum* is an oral anaerobic opportunistic pathogen considered a risk factor for CRC [3]. A recent study showed that the endogenous retroviral-associated adenocarcinoma lncRNA (*EVADR*) was specifically upregulated in metastatic CRC tissues infected by *F. nucleatum*, further conjecturing and confirming that *F. nucleatum* can promote the translation of Snail, Slug, and ZEB1 mRNAs through the lncRNA *EVADR*-YBX1 axis, thus inducing EMT [38] (Figure 2). EMT is the process by which epithelial cells transform into a migratory and invasive mesenchymal phenotype [39]. During this transformation, cells develop loose cell–cell connections and become motile, which results in cancer cell metastasis [40]. EMT is triggered by the dysregulation of the core EMT transcription factors (EMT-TFs), Snail, Slug, and ZEB1, which are essential for maintaining normal epithelial structure [41,42]. A protein called YBX1 regulates mRNA transcription and splicing by binding to DNA/RNA, and it can stimulate the translation of EMT-TFs [43,44]. In summary, *EVADR* directs RNA-binding protein (RBP) YBX1 to recruit its mRNA partners, including Snail, Slug, and ZEB1, to polyribosomes, thereby inducing EMT, ultimately facilitating CRC metastasis. In addition, Chen et al. discovered that *F. nucleatum* infection promoting CRC metastasis could be achieved by upregulating KRT7-AS/KRT7 [17] (Figure 2). The lncRNAs KRT7-AS have been linked to the proliferation and migration of gastric cancer cells in earlier research, and the current study further verified this connection with the metastasis of CRC [17,45]. A downstream target of KRT7-AS that regulates CRC metastasis is KRT7, a type II cytokeratin that can preserve cellular structural integrity and may also increase motile activity [17,46,47]. *F. nucleatum* infection activates the NF-κB pathway, a key regulator of immunity and inflammation whose improper regulation is linked to cancer; activated NF-κB *P*-p65 (activated NF-κB subunit) may upregulate KRT7-AS by increasing the transcriptional activity of KRT7-AS [17,48]. In conclusion, lncRNAs can potentially mediate the role of *F. nucleatum* in promoting CRC metastasis, and these novel mechanisms by which *F. nucleatum* promotes CRC metastasis may offer fresh perspectives on how to treat patients with metastatic CRC.

In addition, *F. nucleatum* can also increase CRC glycolysis by targeting lncRNA enolase1-intronic transcript 1 (ENO1-IT1) and KAT7 histone modification axis, and the activation of glycolysis is one of the causes of chemotherapy failure [49,50,51] (Figure 2). The specific mechanism is that *F. nucleatum* upregulates the binding efficiency of the transcription factor SP1 to the promoter region of lncRNA ENO1-IT1 to activate the transcription of lncRNA ENO1- IT1 and subsequently direct the histone acetyltransferase KAT7 to mediate histone modifications on lncRNA ENO1-IT1 target genes, including ENO1, thereby altering the biological function of CRC, including increasing lactate production, glucose uptake, and cell proliferation that is dependent on glycolysis [49]. KAT7 is a major member of the MYST family of histone acetyltransferases and plays an important role in DNA replication, gene transcription, and embryonic development [52,53,54]. ENO1 can regulate the process related to glycolysis as a key enzyme in the glycolytic pathway [55]. Therefore, targeting ENO1 might improve the sensitivity to chemotherapy and reduce the resistance to oxaliplatin and 5-fluorouracil (5-FU) by inhibiting *F. nucleatum*-induced glycolysis in CRC patients [49]. Given that the combination of oxaliplatin and 5-FU can inhibit DNA synthesis and transcription, thus leading to apoptosis, it is effective to target ENO1 to treat patients with advanced CRC with high levels of *F. nucleatum* [49,56,57]. However, it is still important to determine if, in addition to the enhancement of tumor glycolysis, the method by which *F. nucleatum* upregulates ENO1 to increase drug resistance is connected to other potential processes. Everything considered, *F. nucleatum* activates glycolysis in CRC via targeting the lncRNA ENO1- IT1, which promotes the development of CRC and chemotherapeutic drug resistance in CRC patients, resulting in a poor prognosis.

### 4.2. Enterotoxigenic Bacteroides Fragilis (ETBF) Induces CRC Cells Growth, Proliferation, and Metastasis by Regulating lncRNAs

**Figure 3:**
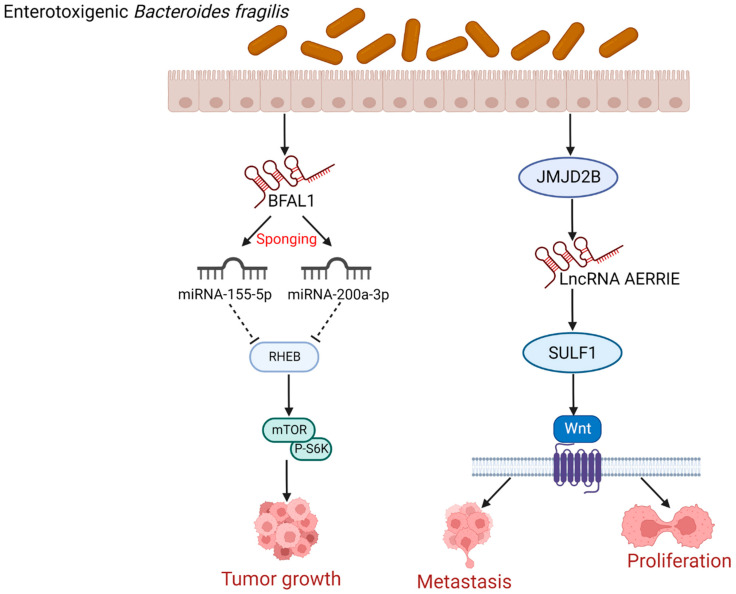
ETBF induces CRC cells growth, proliferation, and metastasis by regulating BFAL1 and AERRIE. BFAL1 competes with miR-155-5p and miR-200a-3p, thus impeding the inhibitory effect of miR-155-5p and miR-200a-3p on RHEB, which upregulates the target RHEB mRNA expression. RHEB can bind directly to the mTOR complex and regulate the mTOR-signaling pathway by phosphorylating the p70 S6K, leading to the activation of the mTOR pathway, thus promoting ETBF-induced CRC cells growth. ETBF can also increase the levels of JMJD2B, and then induce the expression of lncRNA AERRIE, which in turn induces the expression of SULF1. Elevated SULF1 can activate the canonical Wnt pathway, which has been demonstrated to promote the proliferation and metastasis of CRC cells. (Created with BioRender.com). ETBF: Enterotoxigenic *Bacteroides fragilis*; BFAL1: *B. fragilis*-associated lncRNA1; RHEB: Ras homolog enriched in brain; S6K: S6 Kinase; JMJD2B: Jumonji domain-containing protein 2B; lncRNA: long non-coding RNA; SULF1: sulfatase 1.

*Bacteroides fragilis* is an anaerobic bacterium found in the gastrointestinal flora of humans and livestock that produce *Bacteroides fragilis* toxin (BFT), also known as ETBF [58]. The *bft* gene, which encodes BFT, is the main source of pathogenicity for ETBF, which is closely linked to CRC [59,60,61]. In addition to bacterial toxins, researchers have looked into the relationship between lncRNAs and ETBF. A study by Bao et al. reported that *B. fragilis*-associated lncRNA1 (BFAL1) found in CRC tissues was involved in the process of ETBF-induced CRC carcinogenesis by the following mechanism: BFAL1 competes with miR-155-5p and miR-200a-3p, thus impeding the inhibitory effect of miR-155-5p and miR-200a-3p on the Ras homolog enriched in brain (RHEB), which upregulates the target RHEB mRNA expression, leading to the activation of the mTOR pathway for the reason that RHEB is the MTORC1 binding/mammalian target of the rapamycin (RHEB/mTOR) pathway, and ultimately promoting ETBF-induced CRC cells growth [62] (Figure 3). Therefore, BFAL1 may be a promising therapeutic target for ETBF-induced CRC as a mediator of CRC cells growth. Notably, miR-155-5p can encourage the activation of cancer-associated fibroblasts (CAFs) that can promote tumor progression, thus changing the secretory phenotype of CAFs and promoting EMT and metastasis of CRC [63]. In addition, human antigen R (HuR) has been demonstrated to enhance cancer cell growth and migration, while miR-155-5p further positively regulates HuR mRNA expression and cytoplasmic localization in CRC cells, which promotes CRC migration [64]. However, the upregulation of miR-200a-3p inhibits proliferation, migration, and invasion of CRC cells by negatively regulating the forkhead box protein A1 (FOXA1) expression [65]. The various functions of miR-155-5p and miR-200a-3p in CRC are based on their regulatory effects on different downstream molecules. This suggested that the gut microbiome-induced lncRNAs may have the potential to synergize or antagonize miR-155-5p and miR-200a-3p, targeting various downstream molecules to carry out their regulatory roles in CRC, which may become one of the future research directions.

Moreover, there is another potential way by which ETBF might affect CRC. ETBF can also increase the levels of jumonji domain-containing protein 2B (JMJD2B), a potential oncoprotein in the development and progression of CRC [66,67]. In addition, JMJD2B can induce the expression of lncRNA AERRIE, which induces the expression of sulfatase 1 (SULF1) [68]. SULF1 can remove sulfate groups from proteoglycans, and proteoglycan modifications are important for protein interactions and cytokine signaling [69,70]. The activation of the canonical Wnt pathway by SULF1 overexpression has been demonstrated to promote the proliferation and metastasis of CRC cells [8,71] (Figure 3). In summary, ETBF can upregulate JMJD2B to induce lncRNA AERRIE expression and subsequently activate SULF1, thus contributing to CRC cell proliferation and metastasis. This indicated that ETBF holds great promise for research on how it affects CRC by modulating the expression of lncRNAs.

## 5. The Gut Microbiome Metabolic Regulation of CRC Mediated by lncRNAs

### 5.1. Butyrate as the Metabolite of the Gut Microbiome Inhibits the Intestinal Inflammation to Lower the Risk of CRC by Regulating lncRNAs

**Figure 4:**
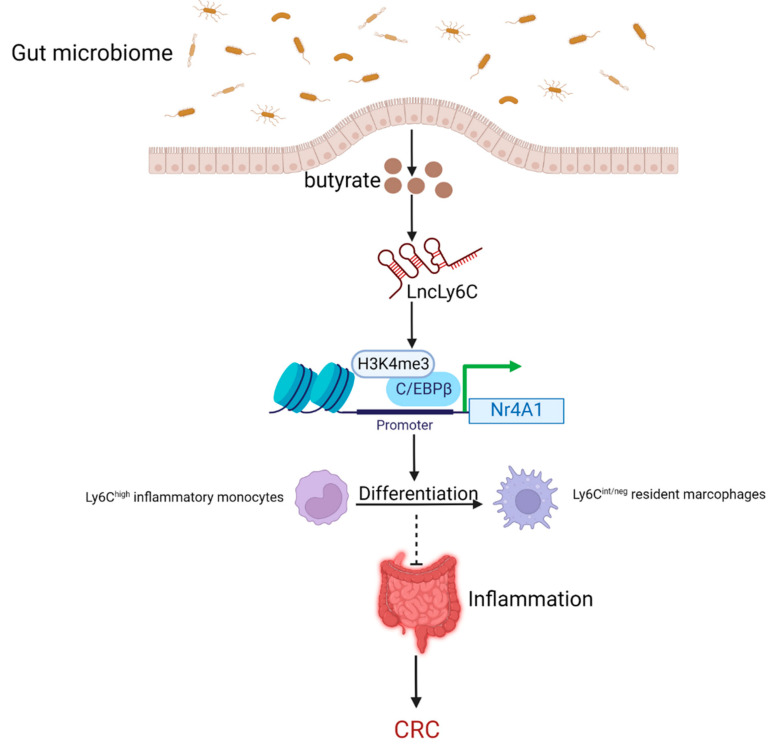
Butyrate as the metabolite of the gut microbiome inhibits the intestinal inflammation to lower the risk of CRC by regulating lncRNA LncLy6C. Butyrate-induced LncLy6C binds to the C/EBPβ and H3K4me3, specifically encouraging the enrichment of C/EBPβ and H3K4me3 marks on the promoter region of Nr4A1, thus promoting the expression of Nr4A1. This promotes the differentiation of Ly6C^high^ inflammatory monocytes into Ly6C^int/neg^ resident macrophages, leading to the inhibition of inflammation. As a result, the risk of developing CAC goes down. (Created with BioRender.com). lncRNA: long non-coding RNA; C/EBPβ: CCAAT/enhancer binding protein β; CAC: colitis-associated colorectal cancer.

In addition to the gut microbiome, which can directly regulate lncRNAs to influence CRC progression, the gut microbiome metabolite butyrate can likewise control the expression of lncRNAs and thus play a significant role in CRC development [37]. Butyrate is a major metabolite produced by the fermentation of dietary fiber bacteria in the gastrointestinal tract. It can help maintain intestinal homeostasis by preventing the expression of pro-inflammatory cytokines and regulating the function of intestinal macrophages by inhibiting histone deacetylases [72,73,74]. The lncRNA LncLy6C, induced by the microbiota metabolite butyrate, binds to the transcription factor CCAAT/enhancer binding protein β (C/EBPβ) and several lysine methyltransferases of H3K4me3, specifically encouraging the enrichment of C/EBPβ and H3K4me3 marks on the promoter region of Nr4A1, nuclear subfamily 4 (NR4A) receptor, which is overexpressed in CRC and plays a central role in controlling cell proliferation, apoptosis, and metastasis [37,75]. This causes the upregulated expression of Nr4A1, promoting the differentiation of Ly6C^high^ inflammatory monocytes into Ly6C^int/neg^ resident macrophages [37] (Figure 4). Ly6C^high^ inflammatory monocytes and Ly6C^int/neg^ resident macrophages represent classical and non-classical macrophages, respectively. Macrophages have been reported to play an important role in intestinal inflammation immunity, EMT and tumor development, and in contrast to Ly6C^high^ macrophages, Ly6C^int/neg^ macrophages are crucial in inhibiting inflammation [37,76,77]. In acutely inflamed colonic tissue, Ly6C^high^ monocytes can migrate to the site of injury, respond to bacterial products, and produce pro-inflammatory effector cells that secrete interleukin (IL)-6 and IL-23, thereby severely promoting colitis [78]. These pro-inflammatory cytokines, produced by inflammatory macrophages, control the activity of intestinal epithelial cells (IECs) by affecting their migration and proliferation, and create a microenvironment conducive to epithelial transformation and tumor development, thus promoting colitis-associated colorectal cancer (CAC) [79]. Therefore, the gut microbiome metabolite butyrate inhibits intestinal inflammation through the pathways mentioned above to lower the risk of CRC.

### 5.2. The Gut Microbiome-Derived Lipopolysaccharide (LPS) Promotes Cancer Cells Migration and Invasion by Regulating lncRNAs

**Figure 5:**
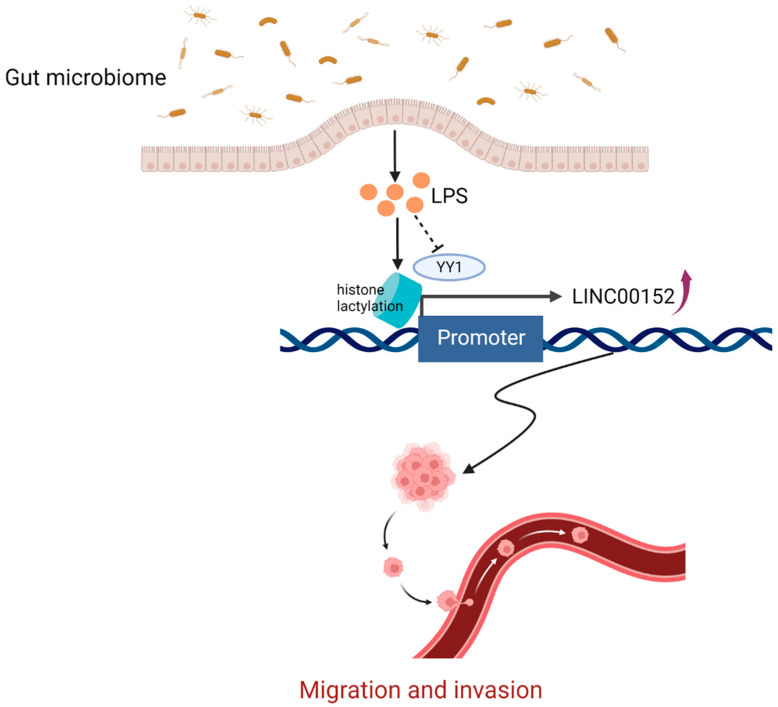
The gut microbiome-derived LPS promotes cancer cells migration and invasion by regulating LINC00152. The bacteria-derived LPS introduces histone lactonization on the promoter of LINC00152 and reduces the binding efficiency of YY1 to LINC00152, thus upregulating the expression of LINC00152. The LPS-induced overexpression of LINC00152 was associated with cancer cells migration and invasion. (Created with BioRender.com). LPS: lipopolysaccharide.

LPS, a characteristic component of the cell wall of gram-negative bacteria, is a typical activator of inflammasomes involved in the development and progression of several diseases, including CRC [80]. A recent study showed that LPS could upregulate the expression of LINC00152, and the overexpression of LINC00152 promotes the migration and invasion of CRC cells [36] (Figure 5). Lactate is a compound produced during the Warburg effect, and lactate-derived histone lysine lactylation has been demonstrated to act as a novel epigenetic modification [81,82]. Histone lactylation can directly stimulate gene transcription from chromatin, which is closely correlated to many pathophysiological processes, including infection and cancer [83]. YY1 is a transcription factor that has been shown to play an important role in the development of cancer, and there is mounting evidence that YY1 is significantly linked to the growth of CRC cells and can be used to predict prognosis in CRC patients [84,85]. It has been demonstrated that YY1 binds to LINC00152 and inhibits its transcription [86]. Therefore, the gut microbiome metabolite LPS introduces histone lactonization on the promoter of LINC00152 and reduces the binding efficiency of YY1 to LINC00152, thus upregulating the expression of LINC00152 [36]. LINC00152, a lncRNA, can promote tumor growth, increase CRC cells migration and invasion, and significantly antagonize 5-FU-induced apoptosis to make CRC cells more resistant to the drug 5-FU by sponging miR-139-5p [87,88,89]. Concludingly, the gut microbiome-derived LPS can upregulate the expression of LINC00152 through the mechanism mentioned above, which negatively influences the development of CRC by encouraging cancer cell invasion and migration.

## 6. Strategies of Preventing and Controlling CRC Based on the Regulatory Role of the Gut Microbiome on lncRNAs

Given the key role of the gut microbiome in CRC development by regulating lncRNAs, some prevention and treatment strategies targeting the gut microbiome have been reported. Among the currently available new therapeutic approaches: traditional probiotics, next-generation probiotics (NGP), and postbiotics can help build healthy gut microbiota in humans as prevention and complementary therapy [90]. Therefore, traditional probiotics can limit the growth of cancer cells by inducing apoptotic pathways (both intrinsic and extrinsic pathways) and can also secrete short-chain fatty acids (SCFAs) that play an active role in improving intestinal barrier function, thereby limiting CRC progression [91,92]. Candidates for NGP in CRC prevention and treatment are bacteria that produce SCFAs, particularly butyrate, capable of exhibiting anti-inflammatory and anticancer properties [90]. Inducing apoptosis in CRC cells, limiting pathogen translocation, repairing the gut barrier, controlling the immune response to inflammation, inhibiting pathogenic bacterial enzymes, exhibiting antimutagenic and antioxidant effects, and lowering intestinal pH and moderating signaling pathways linked to the carcinogenic process are all possible effects of postbiotics [93]. For instance, in cancer immunotherapy, a method for microbe–host interactions involves directly stimulating dendritic cells in lymph nodes via the gut probiotic *Akkermansia muciniphila* to enhance the antitumor activity of immune checkpoint inhibitors in an IL-12-dependent manner [94]. Hence, using probiotics to treat CRC is currently an effective method.

Fecal microbiota transplantation (FMT) as an emerging therapeutic strategy for gastrointestinal illnesses has recently gained popularity. In order to restore intestinal homeostasis, FMT requires introducing healthy gut microbiota into a dysregulated gut microbiome. This is done by transferring feces from a healthy donor into the patient’s gastrointestinal tract [95,96,97]. However, experimental data on the effectiveness of FMT mostly focuses on *Clostridium difficile* infection (CDI) treatment, whereas its use in other gastrointestinal illnesses, particularly in CRC, remains under-investigated, so researchers should focus more on the clinical application in CRC.

Considering that lncRNAs are significant in CRC, we could not dismiss the treatment strategy for CRC that involves controlling lncRNAs. Numerous studies have proved that various lncRNAs, such as WASH5P and CKMT2-AS1, have been available to be potential biotherapeutic targets for CRC, thus limiting the progression of CRC [98,99,100]. The lncRNAs, as emerging biotherapeutic targets for CRC, still have many unanswered questions to be explored, including their role in CRC development and their particular regulatory mechanisms.

## 7. Conclusions

The gut microbes and their metabolites can affect CRC cell growth, proliferation, invasion, metastasis, and drug resistance in CRC patients by regulating lncRNAs due to lncRNAs’ significant roles in many key cellular processes, including apoptosis, proliferation, and differentiation. These findings have great potential in standard clinical trials. First, alterations in the gut microbiome might indicate the development or prognosis of CRC, so there are current approaches, such as probiotic supplements, to improve the gut microbiome to prevent and treat CRC. Second, lncRNAs could be potential targets for CRC diagnosis, prognosis, and treatment, so lncRNAs intervention holds promise as a method to prevent and treat CRC. 

However, current studies on the effects of the lncRNAs-mediated gut microbiome and their metabolites on CRC are limited, and the impacts of multiple gut microbes’ interactions on CRC have not been reported, which should be an explicit topic of interest for future research. Moreover, the interaction of the gut microbiome-induced lncRNAs with other non-coding RNAs, such as microRNAs and circRNAs, in the regulation of CRC also deserves the attention of scientists. Resultantly, it might be possible to develop more methods for CRC prevention and treatment by researching the mechanisms by which lncRNAs mediate the effects of the gut microbiome on CRC. Overall, the regulatory role of the gut microbiome-induced lncRNAs in CRC has great potential and may serve as the foundation for future clinical CRC treatment.

## Supplementary Materials

The following are available online at https://www.mdpi.com/article/10.3390/cancers14235813/s1, Table S1: Mechanisms by which gut microbes and their metabolites impact the development of CRC through the regulation of lncRNAs.

## Abbreviations

CRC	Colorectal cancer
lncRNAs	long non-coding RNAs
TME	tumor microenvironment
EMT	epithelial mesenchymal transition
NF-κB	nuclear factor kappa-B
Pol II	RNA polymerase II
NXF1	nuclear RNA export factor 1
TFs	transcription factors
KRT7-AS	Keratin7-antisense
KRT7	Keratin7
*F. nucleatum*	*Fusobacterium nucleatum*
*EVADR*	endogenous retroviral-associated adenocarcinoma lncRNA
EMT-TFs	EMT transcription factors
RBP	RNA-binding protein
ENO1-IT1	enolase1-intronic transcript 1
5-FU	5-fluorouracil
ETBF	Enterotoxigenic *Bacteroides fragilis*
BFT	*Bacteroides fragilis* toxin
BFAL1	*B. fragilis*-associated lncRNA1
RHEB	Ras homolog enriched in brain
CAFs	cancer-associated fibroblasts
HuR	human antigen R
FOXA1	forkhead box protein A1
JMJD2B	jumonji domain-containing protein 2B
SULF1	sulfatase 1
C/EBPβ	CCAAT/enhancer binding protein β
NR4A	nuclear subfamily 4
IL	interleukin
IECs	intestinal epithelial cells
CAC	colitis-associated colorectal cancer
LPS	lipopolysaccharide
NGP	next-generation probiotics
SCFAs	short-chain fatty acids
FMT	fecal microbiota transplantation
CDI	*Clostridium difficile* infection
