# Supplementary material for: Effects of Long Non-Coding RNAs Induced by the Gut Microbiome on Regulating the Development of Colorectal Cancer
Source: Cancers (Basel). 2022 Nov 25;14(23):5813. doi: 10.3390/cancers14235813 (PMC9735521; doi:10.3390/cancers14235813)
Supplement: Supplementary file 1 [file cancers-14-05813-s001.zip › cancers-2027062-supplementary.pdf]

Table S1. Mechanisms by which gut microbes and their metabolites impact the development of CRC through the regulation of lncRNAs.

Name	LncRNAs	Possible mechanism	Results	References
Fusobacterium nucleatum	*EVADR*	*Fusobacterium nucleatum* upregulates *EVADR*, and elevated *EVADR* directs YBX1 to recruit EMT-related factors, thus enhancing the translation of them thereby inducing EMT.	Metastasis	[38–40]
	KRT7-AS	*Fusobacterium nucleatum* infection activates the NF-κB pathway, and activated NF-κB P-p65 may upregulate KRT7-AS by increasing the transcriptional activity of KRT7-AS, and then activates the downstream target of KRT7-AS, KRT7.	Metastasis	[17,45]
	ENO1-IT1	*Fusobacterium nucleatum* upregulates the binding efficiency of SP1 to the promoter region of lncRNA ENO1- IT1 to activate the transcription of lncRNA ENO1- IT1 and subsequently recruit KAT7 to the promoter of the ENO1 gene to regulate ENO1 transcription via epigenetic modulation.	Drug resistance	[49–51]
Enterotoxigenic *Bacteroides fragilis*	BFAL1	ETBF upregulates BFAL1, and BFAL1 competes with miR-155-5p and miR-200a-3p, thus impeding the inhibitory effect of miR-155-5p and miR-200a-3p on RHEB. RHEB can regulate the mTOR-signaling pathway, leading to the activation of the mTOR pathway.	Growth	[62]
	AERRIE	ETBF increases the levels of JMJD2B, and then induces the expression of lncRNA AERRIE, which in turn induces the expression of SULF1 to activate canonical Wnt pathway.	Proliferation and metastasis	[8,66,68,71]
Butyrate (metabolite)	LncLy6C	Butyrate-induced LncLy6C binds to the C/EBPβ and H3K4me3, specifically encouraging the enrichment of C/EBPβ and H3K4me3 marks on the promoter region of Nr4A1, thus enhancing the expression of Nr4A1. This promotes the differentiation of Ly6Chigh inflammatory monocytes into Ly6Cint/neg resident macrophages.	Reduction of inflammation	[37,76–78]
Lipopolysaccharide (metabolite)	LINC00152	Lipopolysaccharide introduces histone lactonization on the promoter of LINC00152 and reduces the binding efficiency of YY1 to LINC00152, thus upregulating the expression of LINC00152.	Migration and invasion	[36]

References

8. Zhou, L.; Jiang, J.; Huang, Z.; Jin, P.; Peng, L.; Luo, M.; Zhang, Z.; Chen, Y.; Xie, N.; Gao, W.; et al. Hypoxia-induced lncRNA STEAP3-AS1 activates Wnt/beta-catenin signaling to promote colorectal cancer progression by preventing m(6)A-mediated degradation of STEAP3 mRNA. Mol. Cancer 2022, 21, 168.
17. Chen, S.; Su, T.; Zhang, Y.; Lee, A.; He, J.; Ge, Q.; Wang, L.; Si, J.; Zhuo, W.; Wang, L. Fusobacterium nucleatum promotes colorectal cancer metastasis by modulating KRT7-AS/KRT7. Gut Microbes 2020, 11, 511–525. https://doi.org/10.1080/19490976.2019.1695494.
36. Wang, J.; Liu, Z.; Xu, Y.; Wang, Y.; Wang, F.; Zhang, Q.; Ni, C.; Zhen, Y.; Xu, R.; Liu, Q.; et al. Enterobacterial LPS-inducible LINC00152 is regulated by histone lactylation and promotes cancer cells invasion and migration. Front. Cell. Infect. Microbiol. 2022, 12, 913815. https://doi.org/10.3389/fcimb.2022.913815.
37. Gao, Y.; Zhou, J.; Qi, H.; Wei, J.; Yang, Y.; Yue, J.; Liu, X.; Zhang, Y.; Yang, R. LncRNA lncLy6C induced by microbiota metabolite butyrate promotes differentiation of Ly6C(high) to Ly6C(int/neg) macrophages through lncLy6C/C/EBPbeta/Nr4A1 axis. Cell Discov. 2020, 6, 87.
38. Lu, X.; Xu, Q.; Tong, Y.; Zhang, Z.; Dun, G.; Feng, Y.; Tang, J.; Han, D.; Mao, Y.; Deng, L.; et al. Long non-coding RNA EVADR induced by Fusobacterium nucleatum infection promotes colorectal cancer metastasis. Cell Rep. 2022, 40, 111127. https://doi.org/10.1016/j.celrep.2022.111127.
39. Thiery, J.P.; Acloque, H.; Huang, R.Y.J.; Nieto, M.A. Epithelial-Mesenchymal Transitions in Development and Disease. Cell 2009, 139, 871–890. https://doi.org/10.1016/j.cell.2009.11.007.
40. Lv, C.; Yu, H.; Wang, K.; Chen, C.; Tang, J.; Han, F.; Mai, M.; Ye, K.; Lai, M.; Zhang, H. ENO2 Promotes Colorectal Cancer Metastasis by Interacting with the LncRNA CYTOR and Activating YAP1-Induced EMT. Cells 2022, 11, 2363. https://doi.org/10.3390/cells11152363.
45. Huang, B.; Song, J.H.; Cheng, Y.; Abraham, J.M.; Ibrahim, S.; Sun, Z.; Ke, X.; Meltzer, S.J. Long non-coding antisense RNA KRT7-AS is activated in gastric cancers and supports cancer cell progression by increasing KRT7 expression. Oncogene 2016, 35, 4927–4936. https://doi.org/10.1038/onc.2016.25.
49. Hong, J.; Guo, F.; Lu, S.-Y.; Shen, C.; Ma, D.; Zhang, X.; Xie, Y.; Yan, T.; Yu, T.; Sun, T.; et al. F. nucleatum targets lncRNA ENO1-IT1 to promote glycolysis and oncogenesis in colorectal cancer. Gut 2020, 70, 2123–2137. https://doi.org/10.1136/gutjnl-2020-322780.
50. Wang, G.; Wang, J.; Yin, P.; Xu, K.; Wang, Y.; Shi, F.; Gao, J.; Fu, X. New strategies for targeting glucose metabolism–mediated acidosis for colorectal cancer therapy. J. Cell. Physiol. 2018, 234, 348–368. https://doi.org/10.1002/jcp.26917.
51. Ganapathy-Kanniappan, S.; Geschwind, J.-F.H. Tumor glycolysis as a target for cancer therapy: Progress and prospects. Mol. Cancer 2013, 12, 152. https://doi.org/10.1186/1476-4598-12-152.

62. Bao, Y.; Tang, J.; Qian, Y.; Sun, T.; Chen, H.; Chen, Z.; Sun, D.; Zhong, M.; Chen, H.; Hong, J.; et al. Long noncoding RNA BFAL1 mediates enterotoxigenic Bacteroides fragilis-related carcinogenesis in colorectal cancer via the RHEB/mTOR pathway. Cell Death Dis. 2019, 10, 675. https://doi.org/10.1038/s41419-019-1925-2.
66. Liu, Q.-Q.; Li, C.-M.; Fu, L.-N.; Wang, H.-L.; Tan, J.; Wang, Y.-Q.; Sun, D.-F.; Gao, Q.-Y.; Chen, Y.-X.; Fang, J.-Y. Entero-toxigenic Bacteroides fragilis induces the stemness in colorectal cancer via upregulating histone demethylase JMJD2B. Gut Microbes 2020, 12, 1788900. https://doi.org/10.1080/19490976.2020.1788900.
68. Pham, T.P.; van Bergen, A.S.; Kremer, V.; Glaser, S.F.; Dimmeler, S.; Boon, R.A. LncRNA AERRIE Is Required for Sulfatase 1 Expression, but Not for Endothelial-to-Mesenchymal Transition. Int. J. Mol. Sci. 2021, 22, 8088.
71. Ai, X.; Do, A.-T.; Lozynska, O.; Kusche-Gullberg, M.; Lindahl, U.; Emerson, C.P., Jr. QSulf1 remodels the 6-O sulfation states of cell surface heparan sulfate proteoglycans to promote Wnt signaling. J. Cell Biol. 2003, 162, 341–351. https://doi.org/10.1083/jcb.200212083.
76. Bain, C.C.; Mowat, A.M. Macrophages in intestinal homeostasis and inflammation. Immunol. Rev. 2014, 260, 102–117. https://doi.org/10.1111/imr.12192.
77. Li, Y.-H.; Zhang, Y.; Pan, G.; Xiang, L.-X.; Luo, D.-C.; Shao, J.-Z. Occurrences and Functions of Ly6C(hi) and Ly6C(lo) Macrophages in Health and Disease. Front. Immunol. 2022, 13, 901672.
78. Zigmond, E.; Varol, C.; Farache, J.; Elmaliah, E.; Satpathy, A.T.; Friedlander, G.; Mack, M.; Shpigel, N.; Boneca, I.G.; Murphy, K.M.; et al. Ly6C hi monocytes in the inflamed colon give rise to proinflammatory effector cells and migratory anti-gen-presenting cells. Immunity 2012, 37, 1076–1090.
